# Seroprevalence of Brucellosis Among Veterinary Health Personnel in Ernakulam District: A Cross-Sectional Study

**DOI:** 10.7759/cureus.90009

**Published:** 2025-08-13

**Authors:** Harsha Lais, Aswathy Sreedevi, Anil Kumar, Leyanna George, Vijayakumar K

**Affiliations:** 1 Department of Community Medicine, Sree Gokulam Medical College and Research Foundation, Thiruvananthapuram, IND; 2 Department of Community Medicine, Amrita Institute of Medical Sciences and Research Center, Kochi, IND; 3 Department of Microbiology, Amrita Institute of Medical Sciences and Research Center, Kochi, IND; 4 Division of Communicable Diseases, Indian Council of Medical Research, New Delhi, IND; 5 Department of Community Medicine, Health Action by People, Thiruvananthapuram, IND

**Keywords:** brucellosis, enzyme-linked immunosorbent assay, immunoglobulin g, occupational exposure, veterinary personnel

## Abstract

Background: Brucellosis is a zoonotic disease with significant economic and public health implications. Infections in humans are always transmitted through direct or indirect contact with infected animals or their products. A substantial proportion of the Indian population is occupationally exposed to animals, increasing the risk of zoonotic infections such as brucellosis.

Objective: To determine the seroprevalence of brucellosis among veterinary health personnel in Ernakulam district and to analyze the profile of brucellosis cases reported at a tertiary care center over five years.

Methods: A cross-sectional study was conducted among 315 veterinary health personnel from government and private sectors in Ernakulam district, selected through universal sampling. Data were collected using a structured questionnaire, and blood samples were tested for Brucella IgG using the enzyme-linked immunosorbent assay (ELISA). Additionally, a secondary data analysis was done for brucellosis cases reported at a tertiary care center from 2014 to 2019.

Results: Seroprevalence of brucellosis among 315 veterinary health personnel in Ernakulam district was two (0.63%; 95% CI: 0.1-2.3). Among 828 patients presenting with brucellosis-like symptoms at a tertiary care center over five years, 56 (6.76%; 95% CI: 5.06-8.46) were diagnosed with the disease.

Conclusion: Gaps in surveillance, reporting, and screening limit effective control of brucellosis. Enhancing screening of both cattle and humans, along with improving public awareness, food safety, and occupational hygiene, is essential to prevent human infection.

## Introduction

Brucellosis, a neglected zoonotic disease, is a major debilitating illness with significant impact on occupational and public health, particularly in low- and middle-income countries [1]. Close contact with domestic or wild animals increases the risk of acquiring the infection, with casual contact (e.g., touching or proximity) generally considered low-risk, and direct contact with secretions during birthing, slaughter, or specimen collection regarded as high-risk [1]. Consumption of unpasteurized dairy products, such as raw milk and cheese, is also a well-established route of transmission. In India, such exposure accounts for approximately 80% of human cases [2].

Despite its prevalence in many regions, the true extent of human brucellosis is largely unknown. The persistence of animal reservoirs, low physician awareness, limited availability of diagnostic facilities, and the absence of regional databases contribute to the ongoing challenge of controlling this zoonotic disease in India [2].

Although Kerala is projected to have a low frequency of brucellosis cases, active research on the prevalence, distribution, and associated factors of human brucellosis remains limited. In Kerala, brucellosis is not included in the routine surveillance system, but case reports are common in the human and animal health sectors. Notably, a nationwide serological survey by Shome et al. (2019) identified Kerala among eight Indian states where cattle exhibited a true seroprevalence of brucellosis exceeding 5%, indicating a significant presence of the disease in the state's bovine population [3]. Only a few studies have specifically assessed the seroprevalence of human brucellosis among high-risk groups. A study conducted in Thrissur district following a brucellosis outbreak demonstrated a seroprevalence of 2.45% among the general population and 1.14% among veterinary students [4]. Another study in Wayanad among occupationally exposed persons showed a seroprevalence of 1.74% using the standard tube agglutination test [5].

The absence of a single diagnostic test that is both sensitive and specific enough to identify all infection phases or appropriate for all epidemiological scenarios is a diagnostic challenge. However, the enzyme-linked immunosorbent assay (ELISA) offers a more accurate interpretation of diagnostic results, addressing some limitations of the serum agglutination test [6].

As per the 20th Livestock Census (2019), Ernakulam district has approximately 1,05,169 cattle. However, no organized study has been conducted to determine the status of brucellosis among the veterinary health personnel catering to this large population [7]. In recent years, several brucellosis outbreaks have been reported in districts adjacent to Ernakulam. Most recently, in February 2025, the tragic death of a young child in Kerala from brucellosis highlights the urgent need for a One Health approach, which recognizes the interconnection between people, animals, and the environment, and emphasizes early detection, timely interventions, and periodic seroprevalence assessments [8].

Studies in tertiary care centers are rare, as most cases present with non-resolution of symptoms, highlighting the need for sentinel surveillance to better understand and control brucellosis. A retrospective study conducted during 2016-2018 at a tertiary care hospital in southern India reported 94 cases of brucellosis, of which 78.7% were acute, 15.9% subacute, and 5.3% chronic [9]. These findings underscore the under-recognition of brucellosis in its early stages and highlight the value of timely diagnosis.

To bridge this gap, the present study was conducted to estimate the prevalence of brucellosis and its associated risk factors among veterinary health personnel in Ernakulam district, Kerala, and to describe the profile of brucellosis cases reported in a tertiary care center in the district over a five-year period (2014-2019).

## Materials and methods

Study setting

This study was conducted in Ernakulam district and comprised two components: a cross-sectional survey among veterinary health personnel and a retrospective review of brucellosis cases reported from a tertiary care center.

Study population

The cross-sectional component included veterinary surgeons, livestock inspectors, and veterinary attendants working in government institutions such as veterinary hospitals, dispensaries, polyclinics, and subcenters. In the private sector, veterinary handlers were veterinary surgeons and veterinary attendants working in private hospitals with admission facilities in the district. The secondary component included all cases of brucellosis diagnosed using the enzyme-linked immunosorbent assay (ELISA) at the tertiary care center in the district between 2014 and 2019.

Exclusion criteria

Individuals currently undergoing treatment for brucellosis or those treated within the preceding three months were excluded. Additionally, veterinary personnel on extended leave during data collection were excluded from the primary component. For the secondary component, cases with incomplete records or lacking ELISA confirmation were excluded.

Sample size and sampling

Based on a seroprevalence of 16.7% in Bangalore (Agasthya et al.), with 95% confidence and an absolute precision of 5%, the minimum required sample size was calculated as 214 [10]. Universal sampling identified 363 eligible veterinary health personnel from both public and private sectors in Ernakulam District. Of these, 315 consented and formed the final study sample.

Data collection

The data collection was conducted between 2018 and 2019 after obtaining written informed consent from the study participants. A semi-structured questionnaire was used to gather information on their sociodemographic profile, occupational exposure, food habits, and clinical details related to brucellosis. The materials required for blood withdrawal, including disposable needles and Venoject tubes, along with the Brucella IgG ELISA kit (IBL International, Hamburg GmbH, Germany), were procured through the institute’s purchase department following standard indenting procedures. Under aseptic conditions, 5 ml of venous blood was drawn from each participant by trained laboratory technicians. The samples were properly labelled and transported within a few hours to the Department of Microbiology, where they were analyzed for IgG antibodies against Brucella abortus using the Brucella IgG ELISA kit (IBL International GmbH, Germany). The kit reported a sensitivity and specificity of >95%. Participants who tested positive for IgG antibodies were subsequently tested for IgM antibodies to identify recent infections.

To address the secondary objective, the study analyzed the clinical profile of brucellosis cases diagnosed by ELISA at the tertiary care center between 2014 and 2019. After obtaining administrative approval, patient details were retrieved using Medical Record Department (MRD) numbers from the Department of Microbiology. A structured questionnaire was used to extract relevant information, including sociodemographic data, occupational exposure, and commonly reported symptoms among those diagnosed with brucellosis.

Data analysis

The data were tabulated in MS Excel (Redmond, USA) and analyzed using IBM Corp. Released 2014. IBM SPSS Statistics for Windows, Version 20. Armonk, NY: IBM Corp. Quantitative variables were expressed as means and standard deviations, while qualitative variables were presented as absolute numbers and percentages.

Ethical consideration

The study received ethical approval from the Institutional Ethics Committee of Amrita School of Medicine (Dissertation Review/MD/MS/2017/20;09/02/2018). Written informed consent was obtained from all participants before their involvement in the study, which included questionnaire administration and venous blood sampling for brucellosis testing.

## Results

A total of 315 veterinary health professionals in Ernakulam participated in the study. Among them, two (0.63%) tested positive for brucellosis (95% CI [0.1-2.3]). A total of 315 veterinary health professionals in Ernakulam participated in the study. Among them, two (0.63%) tested positive for Brucella IgG antibodies (95% CI [0.1-2.3]); both tested negative for IgM.

The mean age of participants was 42.2 ± 8.7 years. More than half of the respondents were male, 185 (58.7%). The participants' occupational roles included livestock inspectors, 144 (45.7%), veterinary surgeons, 105 (33.3%), and attendants, 66 (21%). Most worked in veterinary dispensaries, 175 (55.6%), followed by hospitals, 53 (16.8%), private hospitals, 27 (8.6%), subcenters, 44 (14%), and polyclinics, 16 (5.1%) (Table 1).

**Table 1:** Distribution of the respondents based on socio-demographic characteristics (n=315)

Variable	Frequency (n=315)	Percentage (%)
Age in years		
≤ 43	160	50.8
> 43	155	49.2
Gender		
Female	130	41.3
Male	185	58.7
Marital status		
Single	31	9.8
Married	278	88.3
Widow	4	1.3
Divorced	2	0.6
Education		
University	37	11.7
Graduate/Postgraduate	199	63.2
Higher Secondary	58	18.4
School Education	21	6.7
Occupation		
Veterinary Surgeon	105	33.3
Livestock Inspectors	144	45.7
Veterinary Attender	66	21.0
Workplace		
Govt Veterinary Dispensary	175	55.6
Govt Veterinary Hospital	53	16.8
Govt Polyclinics	16	5.1
Pvt Veterinary Hospital	27	8.6
Subcenter	44	14.0
Service years		
< 14 years	161	51.1
≥ 14 years	154	48.9

Occupational exposure and preventive practices

A large number of veterinary health professionals reported contact with 272 (86.34%) cattle in the past six months. Over three-quarters, 246 (78.1%), assisted with animal deliveries, and 286 (90.7%) used gloves and other personal protective equipment (PPE) while handling animals. More than half of the participants, 183 (58.1%), reported managing abortion cases within the last year of their service. Most of the participants, 289 (91.7%), were aware of brucellosis, and 225 (71.4%) had attended awareness sessions on the disease. 

Food habits related to brucellosis risk

Most participants, 250 (79.4%), reported having consumed milk at some point in their lifetime, but none reported ever consuming raw milk (Table 2).

**Table 2:** Distribution of respondents according to the occupational exposure and food habits in relation to brucellosis (n=315)

Variable	Frequency (n = 315)	Percentage (%)
Type of animals contacted in the last 6 months		
Cattle	272	86.3
Dog	277	87.9
Cat	243	77.1
Goat or sheep	90	28.5
Pig	13	4.1
Others	10	3.2
Nature of job/activities		
Assisting in deliveries	248	78.7
Collection of specimens	226	71.7
Contact with excreta	199	63.1
Use of personal protective equipment		
Yes	286	90.8
No	29	9.2
Frequency of PPE usage (n = 286)		
Always	146	51
Usually	80	28
Sometimes	60	21
Type of protective measures used (n = 286)		
Gloves	286	100
Eye protection	10	3.1
Face mask	42	13.3
Respirator	42	13.3
Closed footwear	1	0.3
Witnessed any abortion		
Yes	183	58.1
No	132	41.9
Disposal of aborted foetus (n = 183)		
Buried by the owner	154	84.1
Don’t know	29	15.9
Aware of brucellosis		
Yes	289	91.7
No	26	8.3
Received awareness session		
Yes	225	71.4
No	90	28.6
Response if animal is suspected		
Report	256	81.3
Slaughter the animal	15	4.8
Others	2	0.6
Don’t know	42	13.3
Consumption of milk		
Yes	250	79.4
No	65	20.6
Consumption of raw milk (n = 250)		
Yes	0	0
No	250	100

Clinical details related to the disease

Approximately 35 (11.2%) of veterinary health personnel reported experiencing symptoms suggestive of brucellosis, such as unexplained fever, muscle pain, joint pain, or weight loss. Of these, only 17 (48.5%) sought medical treatment for their symptoms. None of the 17 individuals who sought treatment was diagnosed with brucellosis. Additionally, most veterinary health personnel, 305 (96.8%), had never been tested for brucellosis despite their occupational risk.

Secondary data analysis of brucella cases reported at a tertiary care center 

A five-year analysis (2014-2019) of brucellosis cases at a tertiary care center revealed that 56 out of 828 patients (6.76%, 95% CI: 5.06-8.46), presenting with symptoms suggestive of brucellosis (fever, pyrexia of unknown origin, arthralgia, etc.), were serologically confirmed to have the disease. The median age of confirmed cases was 31.0 years (IQR: 14.0-50.8 years). The highest number of cases was observed in the 20-39 years age group (33.9%), followed by 40-59 years (28.6%), <20 years (26.8%), and ≥60 years (8.9%). Males constituted the majority of cases, with 36 (64.3%). While 51 patients (91.1%) were not engaged in livestock-related occupations, five (8.9%) were either butchers, milkers, cattle transport drivers, or veterinary workers. Consumption of raw milk was reported by nine patients (16.07%), highlighting a potential route of transmission. All confirmed cases presented with fever, with 33 (59%) reporting fever alone, 18 (32.1%) reporting fever with joint pain, and five (8.9%) experiencing additional symptoms such as weight loss, headache, and fatigue.

## Discussion

In this study, the seroprevalence of brucellosis among veterinary health personnel in Ernakulam district was 0.63% (2 cases; 95% CI: 0.1-2.3). This is notably lower than the prevalence reported from neighboring districts such as Palakkad and Thrissur, where recent brucellosis outbreaks were reported [11]. A study from central Kerala reported a seropositivity rate of 1.14% among veterinary students and 2.45% among the general population [4]. Similarly, a study in Lakhidi, Palakkad district, revealed a 4.17% seroprevalence among farmers, while veterinarians and para-veterinarians showed zero seroprevalence [5]. These findings suggest that veterinary personnel may have lower seroprevalence due to better preventive measures and strict adherence to safety protocols.

Awareness and reporting practices

In the present study, 289 (91.7%) of veterinary health personnel were aware of brucellosis, and 225 (71.4%) had received awareness training. Additionally, 256 (81.3%) reported that they would notify higher authorities in suspected cases, indicating strong compliance with reporting protocols. A multicentric study across four Indian states in 2021 similarly reported awareness levels of 74% to 85% among veterinarians [12]. In contrast, a 2013 study from southwestern Nigeria reported adequate knowledge of brucellosis in only 30.5% of livestock workers [13]. These trends suggest a gradual improvement in awareness, which may be contributing to the comparatively low seroprevalence observed in this study population.

Animal and environmental context

An unpublished local surveillance report from 2019 indicated zero seroprevalence of brucellosis in 500 cattle samples tested in the region, suggesting low transmission from livestock. This aligns with the low prevalence observed among veterinary professionals. By contrast, national data over the last three decades show a higher incidence range in eastern India (3-56%), followed by the north (3-44%), west (2-13%), and south (0.4-13%) [14].

States such as Punjab, Orissa, Andhra Pradesh, Rajasthan, Maharashtra, Gujarat, Uttar Pradesh, and Goa have reported higher prevalence rates [12]. However, global experiences from Saudi Arabia, Kuwait, and African regions highlight the effectiveness of intersectoral collaboration, vaccination, and livestock import control in reducing brucellosis incidence [14].

Occupational exposure and preventive measures

In this study, 272 (86.4%) of veterinary personnel had direct cattle contact, 63 (20%) owned cattle, 249 (78.7%) assisted with deliveries, 225 (71.4%) collected specimens, 199 (63.1%) reported contact with animal excreta, and 183 (58%) handled aborted fetuses. Despite these exposures, the seroprevalence was low, likely due to the widespread use of PPE.

Notably, 287 (91%) of participants used PPE, primarily gloves. This high compliance likely contributed to the low brucellosis prevalence. In contrast, regions like Punjab have reported inadequate PPE use and a higher risk [15]. Similarly, studies from Egypt, Assam, Uttar Pradesh, and West Bengal have reported PPE scarcity as a contributing factor to disease transmission.

None of our participants consumed raw milk. In Delhi, 57% of peri-urban dairy farmers regularly consumed raw milk [16], while in Kuwait, 77% of brucellosis cases were linked to raw milk consumption [17]. The complete avoidance of raw milk among our study participants is another potential contributor to the low prevalence.

Seroprevalence from hospital-based data

The seroprevalence of brucellosis among patients presenting to a tertiary care center over five years was 6.76% (56 cases; 95% CI: 5.06-8.46). This is consistent with findings from tertiary care centers in Karnataka and Goa, which reported seroprevalence rates of 5.1% and 6.02%, respectively, among patients presenting with pyrexia of unknown origin during the period 2014-2015 [18,19].

Globally, prevalence estimates from hospital settings vary due to regional factors: 14.9% in southwestern Uganda, 23.0% in South Sudan, 3.5% in Tanzania, and 13.7% in Kenya [20].

Age-related findings and routes of transmission

In our study, brucellosis cases were predominantly observed among adults, aligning with previous reports from Bikaner and South India, where most cases occurred in individuals aged 12-60 years [21,22]. This pattern likely reflects higher occupational and environmental exposure among the working-age population, who are more commonly involved in livestock handling, farming, or related activities.

Although raw milk consumption is a recognized route of brucellosis transmission, only nine (16.1%) of the confirmed cases in our study reported this practice. This contrasts with findings from South India and Bikaner, where 72.7% and 86.9% of patients, respectively, reported consuming raw milk [21,22]. The lower frequency in our setting may be attributed to greater awareness and safer dietary practices, possibly supported by Kerala’s high literacy rate.

Occupational risk among brucellosis cases

Among the 56 lab-confirmed brucellosis cases, only five (8.9%) were engaged in livestock-related occupations such as butchers, milkers, cattle transporters, or veterinary workers. This is lower compared to other regions, such as 14% among veterinarians and 26% among slaughterhouse workers in North Karnataka, and 81.1% of seropositive cases reporting occupational exposure in Bikaner [21]. This lower rate of occupational transmission may be attributed to better hygiene practices in the region.

The study has some limitations; it may have missed the participants with the disease on leave, owing to the healthy worker effect, as it was a cross-sectional study. The self-reported data was not rechecked. In an area where the prevalence of brucellosis among animals is low, it is expected that the prevalence among veterinary health personnel will be low. The prevalence of brucellosis, being highly regional, cannot be generalized to all districts of Kerala. 

## Conclusions

The seroprevalence of brucellosis among veterinary health personnel in Ernakulam district was low. Factors such as awareness of the disease, adherence to good hygiene practices, and the use of personal protective equipment were commonly reported among participants and may be associated with the observed low prevalence. However, there remain gaps in the current system, including a lack of proper reporting of suspected cases, limited awareness among grass-roots workers, inadequate screening of cattle and humans, and suboptimal availability of personal protective equipment, which need to be addressed.

Recommendation: Future research should prioritize estimating brucellosis prevalence in both cattle and humans and studying the dynamics of disease transmission. Validating ELISA and other diagnostic methods is also essential to improve accuracy. Given that six out of ten human infectious diseases are zoonotic, a ‘One Health’ approach that integrates human, animal, and environmental health efforts along with adequate government intervention is crucial for effective brucellosis control. Routine screening and surveillance should be implemented for timely detection and response. Training of frontline animal handlers should be strengthened to improve awareness, early reporting, and preventive practices.
